# Advances in Intestinal Glucose Absorption Regulation for Ruminant Energy Efficiency Improvement

**DOI:** 10.3390/ani16040659

**Published:** 2026-02-19

**Authors:** Yan Ye, Xiongfei Zhang, Junhu Yao, Xinjian Lei

**Affiliations:** College of Animal Science and Technology, Northwest A&F University, Yangling 712100, China; 13351590278@163.com (Y.Y.); xiongfeizhang2024@nwafu.edu.cn (X.Z.)

**Keywords:** small intestinal glucose absorption, ruminants, T1R2/T1R3 glucose-sensing pathways, intestinal glucose transport, microbiota

## Abstract

Ruminants can use volatile fatty acids from rumen fermentation for energy, but substantial starch may bypass the rumen and enter into the small intestine under a high-grain diet. In theory, intestinal starch digestion is energetically more efficient than ruminal fermentation. However, ruminants have inherent limits in starch hydrolysis and glucose transport. Small intestinal starch digestion relies on pancreatic α-amylase. Several studies have indicated that functional amino acids (Leu or Phe) may enhance amylase secretion or activity to improve starch digestion. In contrast, strategies to increase glucose absorption efficiency in the small intestine have received less attention. Thus, this review focuses on the effects of diet, ontogeny, environment, and intestinal microbiota on intestinal glucose absorption and their potential mechanisms. The T1R2/T1R3 glucose-sensing pathways, transporting pathways, and related hormones within the small intestine were systematically reviewed. The advantages and limitations of major approaches regarding glucose absorption including portal vein intubation, nutrient perfusion, everted intestinal sacs in vitro, Ussing chamber, brush-border membrane vesicle, D-xylose test, organoid, and nanosensing are also discussed. Importantly, we propose potential strategies to improve small intestinal glucose absorption (e.g., artificial sweeteners and glucagon-like peptide 2-related modulation). Overall, this review summarizes promising regulatory targets to enhance small intestinal glucose absorption and improve energy efficiency in ruminants.

## Simple Summary

Ruminant animals such as cattle and sheep mainly obtain energy from microbial digestion in the rumen. Under a high-grain diet, a large amount of starch can reach the small intestine, where digestion could yield more usable energy. However, ruminants have limited capacity to break starch into glucose and to absorb glucose into the body. An enzyme released by the pancreas is central to starch digestion, and certain dietary amino acids may increase this enzyme and improve starch use. The mechanisms enhancing glucose absorption in ruminants are less understood. This review summarizes how diet, age and development, environmental conditions, and gut microbes influence glucose absorption and describes the key sensing signals, transport steps, and hormone control involved. We also compare common methods used to measure glucose absorption and propose practical strategies to improve it, aiming to boost feed efficiency and livestock productivity.

## 1. Introduction

**Figure 1:**
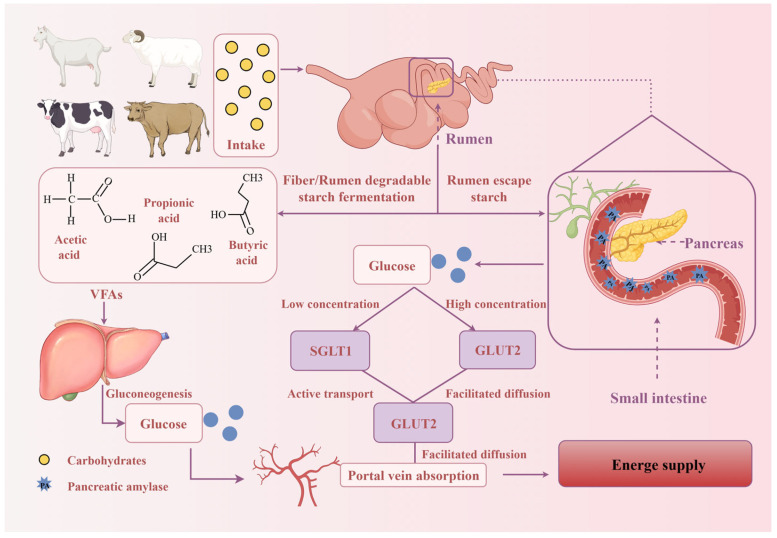
Schematic representation of carbohydrate absorption processes in the rumen and small intestine of ruminants. In ruminants, dietary carbohydrates are fermented by ruminal microorganisms to produce VFAs. Subsequently, they participate in gluconeogenesis and after being converted to glucose in the liver, enter the portal vein. Meanwhile, a portion of starch can escape ruminal fermentation, reach the small intestine, and be hydrolyzed into glucose by pancreatic amylase secreted by the pancreas. At low glucose concentrations in the intestinal lumen, the high-affinity transporter sodium/glucose cotransporter-1 (SGLT1) mediates Na^+^-dependent active absorption; when the luminal glucose concentration increases, glucose transporter 2 (GLUT2) mediates glucose absorption into the portal vein via facilitated diffusion. Both pathways ultimately supply energy to the animal.

Ruminants obtain most of their energy from volatile fatty acids (VFAs) produced by rumen fermentation [1]. Approximately 75% of the VFAs are absorbed into the blood via passive diffusion across the rumen and reticulum wall. The VFAs entering the blood are converted into glucose in the liver (mainly propionic acid), where they participate in gluconeogenesis to supply energy [2]. A notable fraction of dietary starch can escape ruminal fermentation and reach the small intestine. In a high-grain diet, up to 40% of ingested starch may arrive in the small intestine [3]. Moreover, absorption of glucose in the small intestine yields more net ATP than fermenting starch to VFAs in the rumen [3,4,5]. Therefore, shifting carbohydrate digestion from the rumen to the small intestine is beneficial for energy provision. In other words, increasing the level of rumen-escape starch can improve the energy supply efficiency of starch (Figure 1, by Figdraw 2.0).

However, ruminants have physiological limitations in small intestinal digestion and the absorption of carbohydrates [6]. Our previous research has shown that duodenal infusion or feeding ruminal Leu or Phe promotes pancreatic development in goats and increases α-amylase activity, thereby improving starch digestibility in the small intestine [7]. Unlike monogastric animals, ruminants lack intestinal sucrase activity and generally exhibit lower pancreatic and intestinal carbohydrase activities [8]. Moreover, intestinal glucose transporters in ruminants show limited adaptive capacity in response to increased dietary carbohydrate [5,9]. Studies have shown that when the small intestine’s glucose absorption capacity is insufficient, a large amount of undigested starch will enter the hindgut, thereby causing microbial community imbalance, rectal acidosis, colonic mucosal damage, and inflammation [10]. Therefore, optimizing intestinal glucose absorption in ruminants is crucial to enhance their energy utilization and production performance.

This review focuses on the effects of different factors on intestinal glucose absorption and the related mechanisms in ruminants. In addition, we also summarize the established techniques and emerging methods used in this field. This review will provide a theoretical basis and research direction for improving the efficiency of small intestinal glucose absorption by nutritional regulation while maintaining glucose homeostasis in ruminant animals.

## 2. Influence of Multiple Factors on Glucose Absorption in the Small Intestine

### 2.1. Dietary Factors

Dietary fiber, protein, and fat influence intestinal glucose absorption. Chronic fiber supplementation can reduce intestinal glucose absorption and blunt postprandial glycemia in rodents [11]. Especially soluble fiber such as β-glucan is able to help control postprandial glycemia by increasing luminal viscosity, delaying gastric emptying and slowing glucose absorption [12]. Fermentable fibers can further influence absorption indirectly via microbial short-chain fatty acids (SCFAs) and L-cell hormones, which slow gastrointestinal transit and attenuate glycemic excursions [13]. Dietary protein can also modulate glucose uptake [14]. Beyond effects on gastric emptying, amino acids and small peptides stimulate enteroendocrine secretion (e.g., glucagon-like peptide 1(GLP-1), cholecystokinin), thereby altering postprandial motility and the absorptive window. Protein source may matter because digestibility and amino-acid profiles differ and can elicit distinct hormonal responses. A study reported greater promotion of glucose absorption by animal than plant proteins, possibly through differential regulation of intestinal hormone secretion [15].

Dietary fat affects glucose absorption through bile acid-dependent signaling and incretin release. A high-fat diet can alter glucose transport and metabolism in part via gut hormones such as GLP-1 [16]. Evidence from animal models indicates that a high-fat diet may decrease jejunal SGLT1-dependent glucose transport while increasing basolateral GLUT2 expression [17]. More generally speaking, intestinal glucose uptake is mainly mediated by SGLT1 and GLUT2. These transport steps can significantly affect post-meal blood glucose levels due to changes in diet [18].

### 2.2. Developmental and Environmental Factors

Glucose absorption capacity changes markedly during development and is sensitive to environmental stressors. During ontogeny, glucose uptake rises with gestational age and peaks immediately after birth, reflecting rapid adaptation to enteral nutrition and maturation of villus structure, brush-border enzymes, and glucose transporter systems [19]. For example, early weaning in pigs shifts glucose transport toward greater GLUT2-mediated components and alters SGLT1 function in a segment-dependent manner, highlighting that the timing of dietary transition can have durable effects on absorptive physiology [20]. Additionally, hormones and growth factors such as insulin-like growth factor I (IGF-I) and epidermal growth factor (EGF) play pivotal roles in intestinal development, influencing glucose transporter expression and absorptive capacity [21]. Mechanistically, EGF signaling can enhance epithelial differentiation and barrier integrity while upregulating transporter expression and Na^+^/K^+^-ATPase activity, thereby supporting active glucose uptake under inflammatory challenge in porcine models [22]. Environmental factors can also influence the structure and function of the intestine. In addition to the dietary factors mentioned in 2.1, environmental enteropathy is also included. Environmental enteropathy, caused by poor environmental conditions leading to chronic intestinal inflammation and villous atrophy, impairs nutrient absorption, including glucose [23].

### 2.3. Microbial Regulation of Intestinal Glucose Absorption

**Figure 2:**
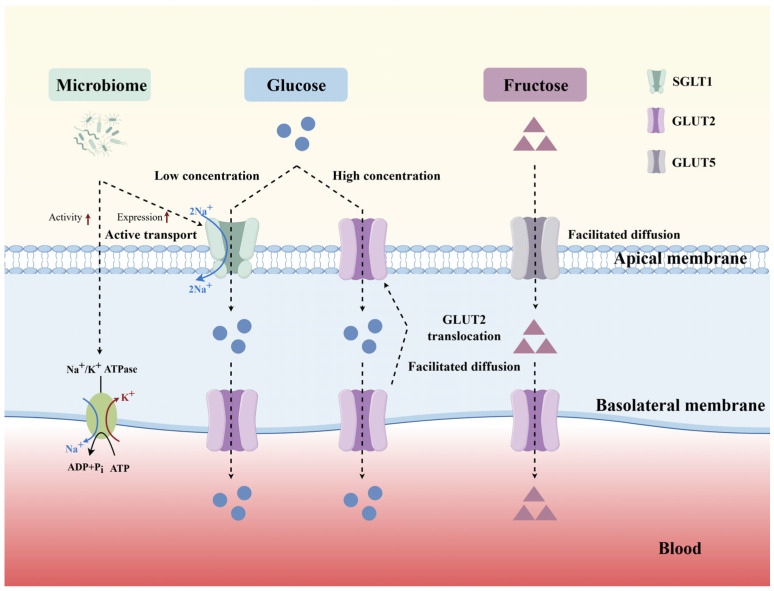
Glucose-related transporters and microbial mechanisms of action. Glucose transport mainly depends on two transporters, SGLT1 and GLUT2. SGLT1 is located in the apical membrane of enterocytes and cotransports one glucose molecule into the cell with two Na^+^ ions through an active transport mechanism driven by Na^+^/K^+^-ATPase when glucose concentrations in the intestinal lumen are low. GLUT2 is responsible for transporting glucose from the cells to the blood. It is typically localized to the basolateral membrane of the intestinal epithelium, where it facilitates the movement of glucose from the intracellular to the blood. When glucose concentrations in the intestinal lumen increase, GLUT2 rapidly relocates to the apical membrane to participate in glucose transport. Fructose enters the cell via facilitated diffusion mediated by GLUT5 and is subsequently transported to the blood through GLUT2 on the basolateral membrane. In addition, the microbiome may affect glucose transport and absorption by increasing SGLT1 expression and Na^+^/K^+^ -ATPase activity. The dotted arrow represents the transport process of glucose, fructose and ions between different parts of the membrane. The blue curve arrow represents the movement of Na^+^ ions. The red curve arrow represents the movement of K^+^ ions. The red short arrow represents an increase.

Existing evidence suggests that the gut microbiome can influence glucose absorption and reshape metabolic signaling through multiple pathways. Cross-species fecal microbiota transplantation showed that microbiota from different pig breeds changed SGLT1 expression and increased the Na^+^/K^+^-ATPase activity after transfer to germ-free mice, as shown in Figure 2 (By Figdraw 2.0) [24]. Microbiota metabolites and bile acid circulation are also involved in the regulation of gut–pancreas–liver axis signaling and intestinal absorption [25,26]. Metformin lowers glycemia in part by increasing microbial diversity, raising SCFAs levels and remodeling bile acid metabolism [27]. Fecal microbiota transplantation from metformin-treated patients increased the abundance of lactic acid bacteria in the small intestinal digesta and upregulated SGLT1 expression, thereby improving whole-body glucose homeostasis [28]. *Lactobacillus* probiotics could also improve glucose metabolism by enhancing the expression of GLP-1 and glucose transporters [29]. Although direct evidence in ruminants is limited, a deeper understanding of microbe-host interactions will help to guide subsequent research.

## 3. Physiological Regulatory Mechanisms of Glucose Absorption in the Small Intestine

### 3.1. Functional Characteristics and Regulation of Glucose-Sensing Receptors

**Figure 3:**
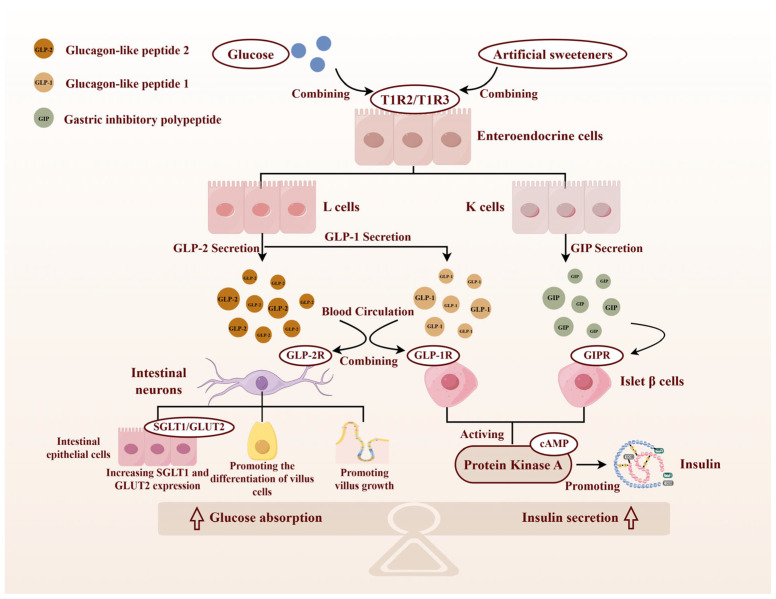
Regulation of glucose-sensing receptor T1R2/T1R3. Binding of glucose or artificial sweeteners to the T1R2/T1R3 receptors on enteroendocrine cells induces the secretion of GLP-1 and GLP-2 by L cells, while simultaneously stimulating the secretion of GIP by K cells. GLP-2 acts on the GLP-2R receptor on enteric nerve cells via blood circulation. It increases SGLT1 expression in intestinal epithelial cells, promotes the differentiation of enterocyte cells, and stimulates the growth of intestinal villi, thereby enhancing glucose absorption in the intestine. At the same time, GLP-1 and GIP activate specific receptors (GLP-1R and GIPR) on islet β cells to activate the cAMP-PKA signaling pathway, thereby promoting insulin synthesis and secretion. They act synergistically to maintain glucose homeostasis. The black solid arrow represents direct biological processes or actions. The upward arrow at the bottom of the figure indicates that both the process of glucose absorption and the secretion of insulin are promoted.

In ruminants, gut glucose sensing is mediated primarily by type 1 taste receptor 2 (T1R2) and type 1 taste receptor 3 (T1R3) expressed on enteroendocrine cells [30]. Glucose or artificial sweeteners can bind to T1R2/T1R3 to regulate glucose absorption and promote the secretion of gastrointestinal hormones such as GLP-1, glucagon-like peptide 2 (GLP-2) and glucose-dependent insulinotropic peptide (GIP) [31] (Figure 3, by Figdraw 2.0).

#### 3.1.1. The Structure of T1R2/T1R3

Sweet taste receptor (STR) is a heterodimer of T1R2 and T1R3 assembled via noncovalent interactions [32]. STR has typical C member of G protein-coupled receptor (GPCR) structural features, contains a Venus flytrap domain (VFTD), cysteine-rich region (CRD) and seven α-helical transmembrane domains (TMDs) [33,34]. Heterodimeric binding sites exist in the TMD region. VFTD is the major ligand binding domain and can specifically recognize sweet substances such as glucose [35]. Glucose binding to VFTD causes closure of the bilobed conformation of this domain. This change induces a global conformational change in T1R2/T1R3. Eventually, the downstream signaling pathways are activated through G proteins [36]. This mechanism underlies sweet chemosensing in the ruminant gastrointestinal tract.

#### 3.1.2. Signal Transduction Pathway of T1R2/T1R3

T1R2/T1R3 signals through two interrelated pathways. One is the inositol trisphosphate (IP3)/diacylglycerol (DAG) pathway that elevates intracellular Ca^2+^. The other involves modulation of adenylyl cyclase (AC) and cyclic AMP (cAMP) levels [37].

T1R2/T1R3 activates α-gustducin upon binding to glucose and produces active G protein α subunits and free β and γ subunits [38]. The activation of the α subunit stimulates intracellular AC. This enzyme produces cAMP, increasing its concentration inside the cell [39]. cAMP can directly induce Ca^2+^ influx or activate protein kinase A (PKA) to inhibit K^+^ efflux. This leads to the release of 5-hydroxytryptamine (5-HT), which activates the taste cell and sends a sweet taste signal to the nerve center. In the IP3/DAG pathway activated by artificial sweeteners, α-gustducin is also activated to produce active G protein α, β and γ subunits. But it is the β and γ subunits that play a role [40]. The β and γ subunits can activate phospholipase C beta 2 (PLC-β2) and catalyze the hydrolysis of phosphatidylinositol 4,5-bisphosphate (PIP2) to IP3 and DAG. IP3 and DAG mediate the release of Ca^2+^ from the endoplasmic reticulum, leading to an increase in intracellular Ca^2+^ concentration. This phenomenon in turn activates protein kinase C (PKC) and transient receptor potential cation channel subfamily M member 5 (TRPM5) [41]. They mediate the closure of the K^+^ efflux channel and the activation of Na^+^ influx channel, respectively, and ultimately transmit sweet taste signals through 5-HT release [42].

#### 3.1.3. T1R2/T1R3 Mediates Gastrointestinal Hormone Secretion

Intestinal T1R2/T1R3 have dual functional regulatory mechanisms. In addition to mediating sweet taste perception in the nervous system, they can also regulate the gastrointestinal hormone secretion network through enteroendocrine cells [43]. Activation of T1R2/T1R3 by glucose or artificial sweeteners stimulates secretion of GLP-1 [44] and GLP-2 [45] from L cells and GIP from K cells. GLP-1 and GIP promote insulin synthesis and secretion by activating specific receptors on islet β cells. GLP-2 significantly enhances intestinal glucose absorption by promoting intestinal villus growth and increasing SGLT1 expression [46] (Figure 3). Previous studies have shown that glucose-stimulated GLP-2 secretion was significantly attenuated when mice were treated with a T1R3-specific inhibitor [47]. More importantly, glucose-induced GLP-1 secretion was completely abrogated in T1R3 knockout mouse models [48]. Thus, T1R2/T1R3-mediated nutrient sensing influences glucose homeostasis mainly by regulating intestinal glucose transporters (e.g., SGLT1/GLUT2), which are described in the next section.

### 3.2. Functional Characteristics and Regulation of Glucose Transporters

#### 3.2.1. Classes of Small Intestinal Glucose Transporters

SGLT1 is a key transporter encoded by the SLC5A1 gene. It contains 14 transmembrane α-helix domains and is mainly located on the apical membrane of villus enterocytes in the small intestine [49]. SGLT1 expression varies along the intestinal axis, being highest in the proximal duodenum and jejunum and decreasing toward the ileum [49].

GLUT2 is a transmembrane protein composed of 524 amino acids. It contains 12 transmembrane α-helical domains. These domains form a typical facilitated diffusion channel. Unlike the high-affinity Na^+^-glucose cotransporter SGLT1, GLUT2 exhibits low affinity but high transport capacity. This property results in significantly better transport efficiency for GLUT2 than for SGLT1 in a high-glucose environment [50].

Glucose transporter 5 (GLUT5) is encoded by the SLC2A5 gene and consists of 12 transmembrane helices. GLUT5 has a higher affinity for fructose than for glucose. Dietary fructose induces GLUT5 expression, thereby enhancing fructose absorption [51]. The distribution of GLUT5 in the small intestine is basically the same as that of SGLT1. The expression of GLUT5 in the duodenum and jejunum is much higher than that in the distal ileum.

#### 3.2.2. Role and Regulatory Mechanism of Small Intestinal Glucose Transporters

SGLT1 is the principal transporter for moving glucose from the intestinal lumen into enterocytes and is often viewed as the gatekeeping step in glucose absorption [52,53,54]. SGLT1 is located on the apical membrane and uses the inward Na^+^ gradient maintained by the basolateral Na^+^/K^+^-ATPase to drive uptake, cotransporting one glucose molecule along with two Na^+^ ions [55] (Figure 2). Ruminant evidence supports a dominant contribution of SGLT1 under physiological conditions. In cattle, at luminal glucose levels of 200 µM, more than 97% of glucose uptake was mediated by SGLT1 [3]. Its high affinity ensures active glucose uptake even when luminal glucose is low [56].

SGLT1 abundance and activity are developmentally regulated. They are highest during lactation, to adapt the increased glucose absorption resulting from lactose breakdown. Subsequently, SGLT1 abundance and activity gradually decreased [57]. Importantly, the adult ruminant intestine retains inducible capacity. Intraduodenal infusion of 30 mM glucose solution for 4 days in adult sheep can increase the glucose transport rate and SGLT1 abundance [57]. Similarly, SGLT1 activity in the jejunum was upregulated in lambs perfused with fructose in the duodenum [58]. Therefore, sugars like glucose can influence the expression of SGLT1 through the intestinal perception mechanism.

GLUT2 is primarily responsible for transporting glucose from absorptive cells into the blood. GLUT2 is normally localized in the basolateral membrane of the intestinal epithelium, transporting glucose across the cell membrane through facilitated diffusion [59]. When luminal glucose rises, GLUT2 is rapidly transferred to the apical membrane to participate in transport [60] (Figure 2). This dynamic regulation process involves a complex signaling cascade. SGLT1-mediated glucose transport triggers membrane depolarization, activates L-type calcium channels and triggers the PKC pathway. This series of events promotes GLUT2 translocation to the apical membrane through cytoskeletal remodeling [61]. The synergistic action of SGLT1 and GLUT2 ensures efficient glucose absorption. GLUT2 also transports fructose and galactose, albeit with lower affinity [62].

However, most studies have not detected significant effects on GLUT2 mRNA or protein in the small intestine under different dietary conditions, including GLUT2 levels in the apical membrane [63,64]. Therefore, it is hypothesized that ruminants mainly exert their effects through SGLT1, while GLUT2 provides a relatively stable exit pathway.

GLUT5 is another transporter expressed in the small intestine and is classically associated with fructose uptake [65]. Fructose presence induces GLUT5 expression [66]. In rodents, GLUT5 mRNA abundance correlates positively with luminal fructose concentration [67]. Compared to monogastric animals, GLUT5 expression in the small intestine of cattle is relatively low, though it is higher in tissues such as the liver and kidneys [68]. However, ruminal supplementation of partially hydrolyzed starch in cattle did not significantly affect GLUT5 expression in all intestinal segments [69]. Thus, GLUT5 plays a relatively limited role in the small intestine of ruminants.

In summary, SGLT1, GLUT2 and GLUT5 together constitute the molecular apparatus for glucose absorption in the small intestine of ruminants.

#### 3.2.3. Mechanisms of Hormonal Regulation

In addition to the regulatory role of transport proteins, the glucose absorption in the small intestine of ruminants is also influenced by hormones, including GLP-2, GLP-1 and insulin. GLP-2 is an intestinotrophic hormone secreted by enteroendocrine L-cells in response to nutrients in the gut [70]. GLP-2 enhances intestinal absorptive capacity by promoting villus growth, crypt cell proliferation, and upregulating transporters [71]. In calves and steers, chronic GLP-2 administration increased mucosal mass, villus height, and brush-border enzyme activities [72,73]. Consistently, transcriptomic profiling in cattle showed that exogenous GLP-2 upregulated genes involved in nutrient metabolism, barrier function, and transporters such as SGLT1 and GLUT2 [72,73,74].

Mechanistically, GLP-2 likely involves the enteric nervous system. GLP-2 receptors are not expressed on enterocytes, but on enteric neurons [75]. When GLP-2 is released, it binds to receptors in the submucosal plexus and triggers a reflex pathway. Research indicates that GLP-2 stimulation causes enteric neurons to release mediators, such as vasoactive intestinal peptide or pituitary adenylate cyclase-activating polypeptide, onto the enterocytes [76]. This cascade upregulates brush-border activity and SGLT1 expression via a cAMP signaling pathway [76]. GLP-2 essentially forms a nutrient-sensing feedback loop. When more nutrients are released in the distal gut, they stimulate the proximal gut to increase absorption capacity. Previous studies have shown that adding an artificial sweetener to ruminant diets activated STR and increased GLP-2 levels. In turn, increased GLP-2 promoted SGLT1 expression and intestinal mucosal growth [76]. Similarly, a study in lambs found that supplementing a high-concentration sweetener in the feed increased endogenous GLP-2 release and accelerated small intestinal development [77]. These findings highlight GLP-2 as a key hormonal regulator linking diet composition to absorptive function.

GLP-1 is co-secreted with GLP-2 by L cells [78]. In ruminants, GLP-1 mainly stimulates insulin secretion and slow gastric emptying [79,80]. GLP-1 increases nutrient absorption time by slowing gastric emptying and intestinal transit [81]. In addition, increasing dietary fat or easily fermentable carbohydrates boosts GLP-1 levels and enhances the postprandial insulin response to glucose [82]. GIP is secreted by enteroendocrine K cells in response to glucose or fat intake [83]. GIP promotes insulin release and may affect intestinal flow or other minor functions [84]. However, its regulatory role in ruminants remains underexplored. The systemic insulin response to absorbed glucose is a key factor [85]. When glucose reaches the bloodstream, insulin levels rise, promoting peripheral glucose utilization [86]. Insulin does not directly regulate intestinal transporter expression, but over time, higher insulin levels may affect the gut’s metabolic state. In one study, increased net glucose absorption across the portal vein was accompanied by higher insulin concentrations in cows [82].

In summary, GLP-2 is a crucial positive regulator of intestinal glucose absorption capacity in ruminants, promoting a larger gut surface and higher transporter levels [72]. Using GLP-2 through diet or drugs shows promise in promoting enteral nutrient absorption. Hormones like GLP-1, GIP and insulin may also regulate the absorptive environment.

## 4. Research Methods and Techniques Progress

### 4.1. In Vivo Experimental Techniques

#### 4.1.1. Nutrient Disappearance and Portal Absorption

Small intestinal absorption can be estimated using duodenal and ileal cannulation. Administer a fixed amount of starch. Collect the digesta that reaches the ileum, and estimate how much carbohydrate the small intestine absorbed by calculating the difference between the amount fed and the amount collected from the ileum [87]. A more direct approach is portal vein catheterization, which quantifies the net portal appearance of glucose into the blood [88]. In ruminants, catheters and flow probes are placed on the portal vein and artery to measure nutrient flow. By sampling blood from the portal vein and artery, one can calculate net absorption as (portal–arterial concentration difference) × (blood flow). After a high-starch diet or an intraduodenal glucose infusion, portal glucose concentrations rise and the absorbed moles of glucose can be quantified [89]. A study supplied butyric acid to the posterior segment of the gastrointestinal tract of lambs and found that the nutrient flux in the portal vein changed and butyric acid treatment enhanced glucose metabolism [90]. This gold-standard method reflects near-physiological conditions. However, this approach integrates flux from the entire intestine and cannot localize segmental contributions. In addition, it is costly and requires specialized facilities, strict surgical preparation, and intensive postoperative care.

#### 4.1.2. D-Xylose Absorption Test

In veterinary research, the D-xylose absorption test is used to assess small intestinal function. D-Xylose is a sugar that is readily absorbed and only minimally metabolized. After administering a dose of D-xylose orally, blood or urine concentrations are measured to evaluate absorptive capacity [91]. A healthy intestine absorbs xylose, causing an increase in blood xylose. But if the intestine is damaged, it can lead to malabsorption of xylose [92]. D-xylose absorption test has been applied in calves and adult cattle to diagnose malabsorption and determine the condition of intestinal damage [93,94].

### 4.2. In Vitro Experimental Techniques

#### 4.2.1. Intestinal Loop Techniques

After anesthesia, a segment of the intestine is exteriorized and isolated while preserving its mesenteric blood and nerve supply. Both ends are ligated to create a closed intestinal loop. A known concentration of glucose solution is injected into the loop. After a defined incubation period, the luminal fluid is collected. The glucose concentration in the collected solution is measured. The glucose absorption rate per unit time and length is calculated from the reduction in glucose [95]. This approach provides direct estimates of absorptive capacity, but it is invasive and requires anesthesia or euthanasia, so hormonal and neural regulation may not fully reflect the physiological state. Meanwhile, a study has provided a detailed description of the intestinal loop technique for establishing a cannulated intestinal loop model in the sheep’s ileum, which can be used to target drug administration or perfusion in different intestinal segments and to measure local responses [96]. Therefore, this technology can also be applied in models related to intestinal physiology, immunity or targeted processing.

#### 4.2.2. Everted Gut Sacs

The everted gut sac is a widely used ex vitro model for studying intestinal transport and the absorption of nutrients and drugs. It was applied in early ruminant studies to investigate the uptake of intestinal compounds [97,98]. A segment of jejunum is removed from a sacrificed animal, turned inside-out, and tied off into a small sac. The sac is incubated in buffer containing a known glucose concentration. After incubation, the fluid inside the sac is analyzed to determine how much glucose moved from the exterior to the interior. Tissue viability is a key limitation. Under physiological conditions, intestinal segments remain viable and metabolically active for only about two hours. Another drawback is that the muscular layer cannot be removed easily, which can underestimate apparent transport and can lead to nonspecific binding of compounds to muscle cells [99].

#### 4.2.3. Ussing Chamber Assays

The Ussing chamber is a classic in vitro technique for measuring electrogenic ion and nutrient transport across epithelial tissues. In glucose absorption studies, it provides real-time, quantitative readouts of transporter activity and barrier function. A sheet of intestinal mucosa is mounted between two half-chambers to create mucosal side and serosal compartments with defined bathing solutions. Because SGLT1 mediates Na^+^ -coupled glucose uptake, adding glucose to the mucosal side increases the short-circuit current, and this response is blocked by SGLT1 inhibitors, allowing quantification of SGLT1-dependent transport [17]. The Ussing chamber can also be used to measure transepithelial movement of radiolabeled glucose. In ruminant tissue, studies in ovine rumen epithelium showed that GLP-2 modulates ion transport [100]. Furthermore, a study employed Ussing chamber assays to measure the Ca^2+^ absorption rate on the intestinal tissues of sheep and goats, thereby evaluating the transmembrane absorption characteristics of calcium ions in the rumen of ruminants [101]. The advantage of the Ussing chamber is fine control and the ability to assess mechanisms. Limitations include labor-intensive setup, the need for small viable tissue pieces, and limited ex vivo viability of only a few hours [102].

#### 4.2.4. Brush-Border Membrane Vesicles (BBMVs)

The BBMV technique is a classic in vitro method for quantifying apical transporter activity in small intestinal epithelial cells. Membrane vesicles are isolated from the intestinal mucosa by sucrose density gradient centrifugation. Transport of specific substrates is then measured in a physiologically relevant buffer using radiolabeled or fluorescent tracers. In ruminants, BBMVs have been used to quantify SGLT1 activity. Researchers isolated BBMVs from the small intestines of sheep or cattle and measured glucose uptake in the presence or absence of Na^+^, which allowed them to determine the contributions of SGLT1- and GLUT2-mediated transport [3]. The method has also supported numerous comparative studies across species [103]. For instance, BBMVs have been used to assess both the activity and abundance of SGLT1 in herbivores, including roughage-fed ruminants [104]. A key strength of the BBMVs approach is that it measures transporter function in isolation. But it requires fresh tissue and careful standardization of membrane preparation.

### 4.3. Emerging Experimental Techniques

In recent years, nanosensor technologies have become powerful tools for studying intestinal glucose absorption. These nanoscale systems enable real-time, high-sensitivity monitoring of glucose dynamics. For example, a fluorescent nanosensor using phenylboronic acid-functionalized reduced graphene oxide coupled to a diol-modified fluorescent probe has been developed for glucose detection [105]. Single-nanowire glucose sensors also allow real-time monitoring of intracellular glucose levels [106]. Nevertheless, nanosensor readouts may be influenced by biocompatibility and long-term stability issues. Since 2009, long-term in vitro intestinal organoids have advanced stem cell biology, disease modeling, and personalized medicine [107]. Murine intestinal organoids have been used to assess nutrient transport and sensing [108]. 3D organoids have been validated for studying nutrient and drug transport and metabolism using uptake assays and metabolomics, providing a robust platform for intestinal research [109]. Organoids also offer a superior epithelial model for evaluating drug bioavailability, supporting drug development and enabling toxicology testing [110,111]. However, organoid models lack full tissue complexity, such as vascular, immune, and microbial components, and these factors should be considered when interpreting the results.

In summary, each research method has distinct strengths and limitations. In vivo approaches capture whole-animal responses but make it difficult to isolate specific mechanisms. In vitro techniques allow targeted study of intestinal function yet omit systemic influences. Therefore, combining complementary methods is often necessary to reach reliable conclusions.

## 5. Conclusions

Intestinal glucose absorption is regulated by multifactorial influences such as the diet, developmental stage, environment and microbiome. Mechanistically, uptake in the small intestine is mainly dependent on a two-step transport model: SGLT1 on the apical membrane mediates sodium-coupled glucose entry, while GLUT2 on the basolateral membrane facilitates efflux into the circulation. Chemosensing pathways and enteroendocrine hormones also provide plastic regulatory windows for intestinal absorption. Intestinal T1R2/T1R3 senses glucose or artificial sweeteners, promoting the secretion of GLP-2 and GLP-1. GLP-2 augments absorption by upregulating SGLT1 expression and promoting intestinal development through enteric neural reflexes. GLP-1 and GIP primarily affect systemic glucose homeostasis by regulating gastric emptying and pancreatic islet function. Furthermore, the various research methods systematically elaborated in the text provide a solid foundation for translating theoretical insights into practical applications. From a practical perspective, the most promising strategies are: using artificial sweeteners to activate the T1R2/T1R3–GLP-2 axis; targeted amino acid supplementation to support mucosal integrity; and interventions amplifying endogenous GLP-2 signaling. Future studies should prioritize elucidating the microbiome-mediated mechanisms governing small intestinal glucose homeostasis in ruminants.

## Data Availability

No new data were created or analyzed in this study. Data sharing is not applicable.

## Abbreviations

The following abbreviations are used in this manuscript:

GLP-1	Glucagon-like peptide 1
IGF-I	Insulin-like growth factor I
EGF	Epidermal growth factor
SCFAs	Short-chain fatty acids
SGLT1	Sodium/glucose cotransporter-1
T1R2	Type 1 taste receptor 2
T1R3	Type 1 taste receptor 3
GLP-2	Glucagon-like peptide 2
GIP	Glucose-dependent insulinotropic peptide
STR	Sweet taste receptor
GPCR	C member of G protein-coupled receptor
VFTD	Venus flytrap domain
CRD	Cysteine-rich region
TMD	Transmembrane domains
IP3	Inositol 1,4,5-trisphosphate
DAG	Diacylglycerol
AC	Adenylyl cyclase
cAMP	Cyclic AMP
PKA	Protein kinase A
5-HT	5-hydroxytryptamine
PLC-β2	Phospholipase C beta 2
PIP2	Phosphatidylinositol 4,5-bisphosphate
PKC	Protein kinase C
TRPM5	Transient receptor potential cation channel subfamily M member 5
BBMVs	Brush-border membrane vesicles
